# Bilateral osteoporotic bone marrow defects of the mandible: a case report

**DOI:** 10.1186/1746-160X-8-22

**Published:** 2012-08-08

**Authors:** Diego Mauricio Bravo-Calderón, Denise Tostes Oliveira, Wagner Humberto Martins dos Santos

**Affiliations:** 1 Department of Stomatology, Area of Pathology, Bauru School of Dentistry, University of São Paulo, Alameda Octávio Pinheiro Brisolla, 9–75, Bauru, 17012-901, SP, Brazil; 2 Private Practice, Rua Almirante Barroso, 1433, Ji-Paraná, 76900-079, RO, Brazil; 3 Bauru School of Dentistry, Department of Stomatology, Area of Pathology, Alameda Octávio Pinheiro Brisolla, 9–75 CEP 17012–901, Bauru, São Paulo, Brazil

**Keywords:** Mandible, Bone marrow pathology, Radiography

## Abstract

Osteoporotic bone marrow defect of the jaws has been reported as a poorly demarcated radiolucency that affect mainly posterior mandible of middle-aged woman. The incidence of this condition is not exactly established and its pathogenesis remains unknown. An additional unusual case of osteoporotic bone marrow defects occurring bilaterally in the mandibular edentulous regions of a 32-year-old white woman is presented reinforcing its diagnostic criteria and histopathological findings.

## Background

In adult life the presence of hematopoietic marrow in the jaws is usually restricted to the angle of the mandible, the maxillary tuberosity and the condylar process [1,2]. Osteoporotic bone marrow defect is a radiolucent area that corresponds to the uncommon presence of hematopoietic tissue found in others regions of the jaws [2]. The defect is generally asymptomatic and is discovered incidentally during radiographic analysis. Radiographically, it is a localized radiolucency that varies in size, shape, trabeculae and border definition [2-4].

Since the osteoporotic bone marrow defect is rarely included in the differential diagnosis of radiolucent lesions of the jaws, the knowledge of the clinical, radiographic and histopathological characteristics in association with an accurate examination, are mandatory, in order to distinguish it from other most common intrabony lesions as odontogenic tumors or cysts, pseudocysts or primary or metastatic malignancies.

This paper describes an unusual case of bilateral bone marrow defects and discusses the characteristics and etiopathogeny of this condition.

## Case report

**Figure 1:**
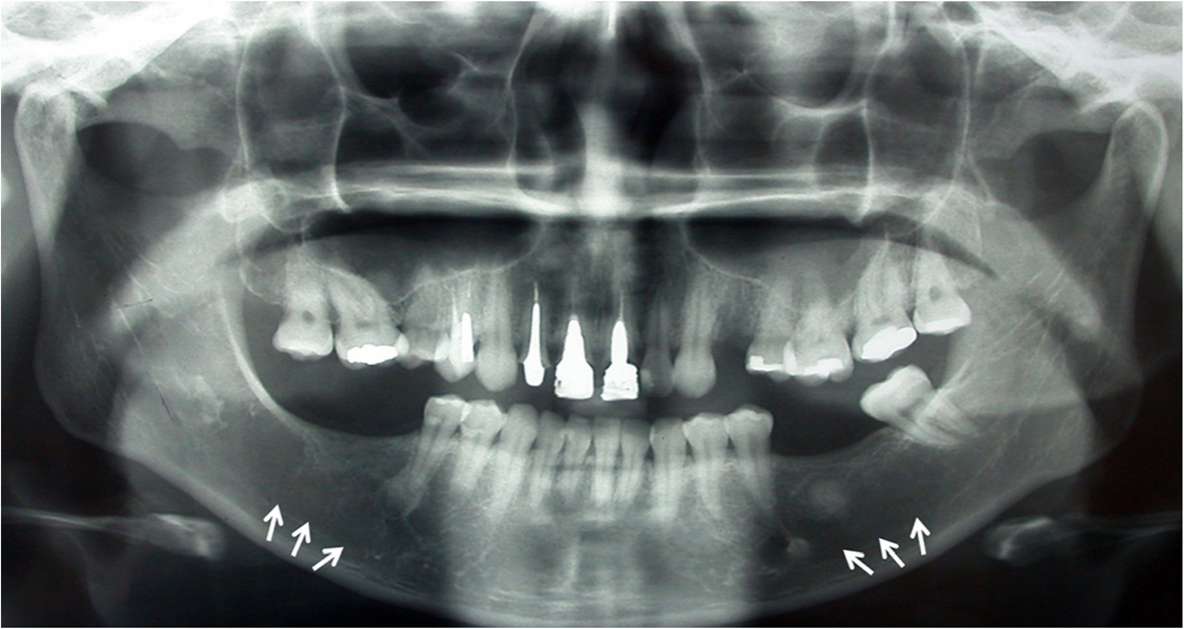
Panoramic radiography demonstrating ill-defined radiolucent areas located bilaterally in the molar edentulous regions (white arrows).

**Figure 2:**
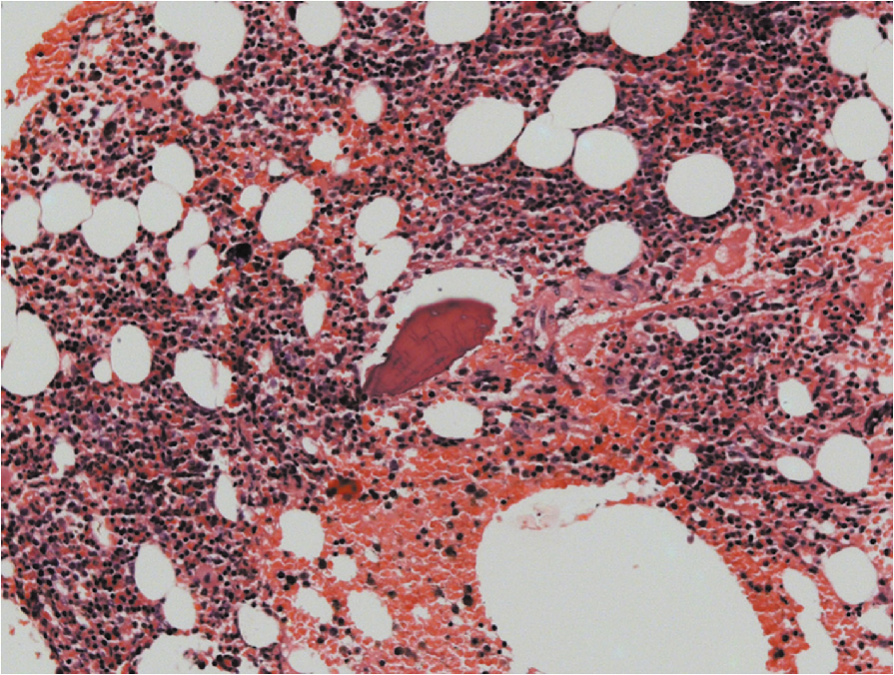
**Histopathologic findings.** Normal hematopoietic cells, fat cells and bone trabeculae. (Hematoxylin and eosin. Original magnification X200).

**Figure 3:**
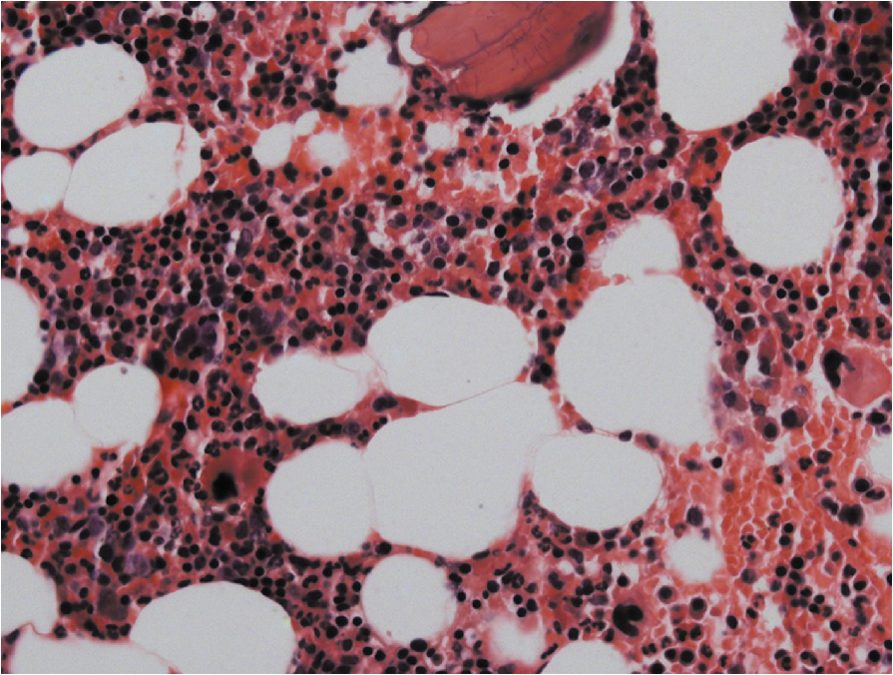
**Microscopic details of the bone marrow.** Erythroid, granulocytic, monocytic and lymphocytic series are illustrated, as well as megakaryocytes. (Hematoxylin and eosin. Original magnification X400).

A 32-year-old white woman was referred to private dental clinic for routine prosthodontic treatment. Intraoral examination revealed healthy mucosa and there was not any sign of infection. Her past medical history was unremarkable. Panoramic radiography of the jaws showed 4 cm x 3 cm radiolucencies with quite ill-defined and irregular borders located bilaterally in molar edentulous regions (Figure 1). Two small sclerotic flecks were associated with left radiolucent area. The lesions were asymptomatic and no expansion of the cortical jawbone was detected. A provisional diagnosis of odontogenic cyst or tumor was made and focal osteoporotic bone marrow defect was considered as a differential diagnosis based on age, site, clinical and radiographic findings. Under local anesthesia, a biopsy specimen was performed from the both edentulous molar regions and the tissues submitted to the Bauru School of Dentistry Oral Pathology Biopsy Service of the University of São Paulo. The histopathological examination revealed normal hematopoietic bone marrow characterized by erythroid, granulocytic, monocytic and lymphocytic series (Figure 2). In addition, megakaryocytes, fat cells and bone trabeculae were also observed (Figure 3). Abnormal morphology of the hematopoietic cells or malignant cells was not seen. The histopathological findings confirmed the diagnosis of osteoporotic bone marrow defects of the mandible.

## Discussion

The osteoporotic bone marrow defect of the jaws has been reported as an unusual radiolucency often detected fortuitously in the posterior mandible of the middle-aged woman [1,4-6]. Radiographically, this radiolucency varies in size from several millimeters to centimeters, the shape and borders are ill defined [2,3,6]. The condition frequently occurs in an edentulous region where tooth extraction was previously performed [1,4]. Clinically, some osteoporotic bone marrow defects are multifocal, others bilateral and few of them are symptomatic [1-4,7].

The present case confirms many of the findings described above but bilateral mandibular involvement of the osteoporotic bone marrow is not frequently found in the literature [1,7-9]. Of the 20 cases of osteoporotic bone marrow defect described by [Makek and Lello 1] only one was bilaterally located in the molar areas of the mandible and we found one case reported, in Brazil, of this condition involving bilateral edentulous mandibular regions of a 54-year-old-woman [8]. According to study conducted by Bouquot and collaborators [7] based in the literature review and report of 596 new cases, the bilateral occurrence of osteoporotic bone marrow defect within the jaws affected 3 % of patients.

There are many reported about this issue but the real incidence of osteoporotic bone marrow defect is not accurately established, probably due to the fact that most cases, including our case reported, are asymptomatic and discovered in radiographic exam for diagnosis of oral conditions [1,6,8,9]. In addition, larger studies about this osteoporotic defect fail to mention the number of cases with available radiographs [2].

Previous osteoporotic bone marrow defects documented in the English literature [1-5] indicate that, frequently, its radiographic appearance may be confused with other intraosseous pathologic conditions. As described in the present report, the bilateral radiolucent areas with indistinct margins suggested the presence of the mandibular benign cysts or tumors. Based on age, site, clinical and radiographic findings, the osteoporotic bone marrow defect was considered as a differential diagnosis. The final diagnosis of osteoporotic bone marrow defect should be established on microscopic features rather than clinical parameters [2,4]. Microscopically, the presence of hematopoietic marrow composed of erythroid, granulocytic, monocytic and lymphocytic series, as well as megakaryocytes associated with fatty marrow is required for diagnosis of this condition [1,4-6]. In the case reported, the association of histopathological and clinical features allowed the precise diagnosis of the osteoporotic bone marrow defect.

Regarding the etiopathogenesis of osteoporotic bone marrow defect of the jaws, three major theories have been proposed. First, it was thought in the possible persistence in adult life of red embrionary marrow that had no conversion to fatty marrow [2]. However, many authors [1,2] have ruled out this suggestion because if it were possible, this condition would have similar distribution between adults and adolescents or children, but it affect frequently patients in the fourth to six decade of life. Second theory proposed that the compensatory bone marrow hyperplasia were caused by increased functional demand for erythrocytes that occurs in systemic diseases including sickle cell anemia [2,9]. However, of the series of cases reported in the literature only few found relationship between osteoporotic bone marrow defects and anemia [2], and only Sanner and Ramin [9] documented a case of a 48-year-old man with a bilateral osteoporotic bone marrow defect associated to sickle cell anemia. In our case reported, the haematological examination no revealed any systemic alteration in the patient. The third hypothesis regards to deficiencies in bone repair in areas of previous trauma such as a tooth extraction [2], where the transient ischemia induce the osteoporotic bone marrow defects [4]. According to literature, yellow bone marrow contains mesenchymal progenitor cells capable of producing haematopoietic microenvironment [10] and these cells were recently isolated from mandibular marrow aspirates [11]. In this context, this theory seems to be the most feasible in the present case, due to the close proximity of the two lesions with edentulous regions.

In conclusion, this case reported of uncommon bilateral osteoporotic bone marrow defects reinforces that, although the treatment is not required, the radiographic findings are not sufficient to establish an exact diagnosis and histopathological examination is indicate for suspected radiolucencies in the jaws.

## Consent

Written informed consent was obtained from the patient for publication of this Case report and any accompanying images. A copy of the written consent is available for review by the Editor-in-Chief of this journal.

## Competing interests

The authors declare that the article-processing charges were supported by Pós-graduação FOB-USP – Proap/Capes and Conselho Nacional de Desenvolvimento Científico e Tecnológico (CNPq – grant #142790/2011-7).

## Authors’ contributions

DMB-C and DTO prepared the case report, discussion and manuscript, WHMS collected the clinical data and performed the surgical procedures, DMB-C and DTO did the histopathological analysis. All authors have read and approved the final manuscript.
